# The Modulation of Chaihu Shugan Formula on Microbiota Composition in the Simulator of the Human Intestinal Microbial Ecosystem Technology Platform and its Influence on Gut Barrier and Intestinal Immunity in Caco-2/THP1-Blue™ Cell Co-Culture Model

**DOI:** 10.3389/fphar.2022.820543

**Published:** 2022-03-15

**Authors:** Ling Liu, Yi Lu, Chao Xu, Haitao Chen, Xuanying Wang, Yijie Wang, Biyu Cai, Bing Li, Lynn Verstrepen, Jonas Ghyselinck, Massimo Marzorati, Qinghua Yao

**Affiliations:** ^1^ Department of Integrated Chinese and Western Medicine, The Cancer Hospital of the University of Chinese Academy of Sciences (Zhejiang Cancer Hospital), Institute of Basic Medicine and Cancer (IBMC), Chinese Academy of Sciences, Hangzhou, China; ^2^ Department of Clinical Nutrition, The Cancer Hospital of the University of Chinese Academy of Sciences (Zhejiang Cancer Hospital), Institute of Basic Medicine and Cancer (IBMC), Chinese Academy of Sciences, Hangzhou, China; ^3^ Second Clinical Medical College, Zhejiang Chinese Medical University, Hangzhou, China; ^4^ Leuven Health Technology Centre China Centre, Hangzhou, China; ^5^ ProDigest BV, Technologiepark, Zwijnaarde, Belgium; ^6^ Center of Microbial Ecology and Technology (CMET), Faculty of Bioscience Engineering, Ghent University, Ghent, Belgium; ^7^ Key Laboratory of Traditional Chinese Medicine Oncology, Zhejiang Cancer Hospital, Hangzhou, China; ^8^ Key Laboratory of Head and Neck Cancer Translational Research of Zhejiang Province, Hangzhou, China

**Keywords:** chaihu shugan formula, gut microbiota, gut barrier, intestinal immunity, SHIME^®^ platform, human

## Abstract

The traditional Chinese medicine (TCM)–Chaihu Shugan Formula (CSF), consisting of several Chinese botanical drugs like Bupleurum, is derived from the ancient Chinese pharmacopeia. It has been used for more than thousands of years in various suboptimal health statuses and diseases induced by chronic stress based on empirical therapy. Recent studies confirm the role of CSF in the development of many diseases, including depression, stress-induced hepatic injury and tumors. However, little has been known about the mechanisms behind the health effects of CSF. Here, we investigate the influence of CSF on the modulation of the simulated colonic microbiota of five healthy donors, gut barrier integrity, and intestinal immunity by combining the simulator of the human intestinal microbial ecosystem (SHIME^®^) technology platform with co-culture of intestinal and immune cells. This approach revealed that CSF stimulated the production of SCFA (acetate, propionate and butyrate) across donors while significantly lowering the production of branched SCFA (bSCFA). In terms of community composition, CSF stimulated a broad spectrum of health-related Bifidobacterium species, which are potent acetate and lactate producers. At the same time, it lowered the abundance of opportunistic pathogenic *Escherichia coli*. Later, we explore the effect of colonic fermentation of CSF on the gut barrier and intestinal immunity in the Caco-2/THP1-blue™ cell co-culture model. Based on the study using SHIME technology platform, CSF showed protective effects on inflammation-induced intestinal epithelial barrier disruption in all donors. Also, the treatment of CSF showed pronounced anti-inflammatory properties by strongly inducing anti-inflammatory cytokines IL-6 and IL-10 and reducing pro-inflammatory cytokine TNF-α. These findings demonstrate a significant modulatory effect of CSF on intestinal gut microbiota. CSF-microbial fermentation products improved the gut barrier and controlled intestinal inflammation.

## Introduction

Traditional Chinese medicine (TCM), an empirical medicine that developed for thousands of years, has established its own medical system differing from modern medicine and has been widely used in many Asian countries, especially in China (Cheung, 2011; Xu et al., 2019). Emerging studies show that it has held great potential for health maintenance, clinical management of various diseases, and new drug discovery (Ma et al., 2013; Sucher, 2013; Xiang et al., 2019; Yang et al., 2020). Currently, TCM is becoming more frequently used in countries in the West. Chaihu Shugan Formula (CSF) is a traditional Chinese medicinal herbal formula, which comprises of several Chinese botanical drugs, including *Bupleurum chinese* *DC* (Chinese name Chai-hu), *Pericarpium citri reticulatae* (Chinese name Chen-pi), *Ligusticum chuanxiong Hort* (Chinese name Chuan-qiong), *Rhizoma cyperi* (Chinese name Xiang-fu), *Fructus aurantii* (Chinese name Zhi-ke), *Radix paeoniae alba* (Chinese name Bai-shao) and *Glycyrrhiza uralensis Fisch* (Chinese name Gan-cao). CSF was firstly recorded in the Chinese pharmacopeia called “Jing Yue Quan Shu”, written by a famous physician named Jingyue Zhang in 1,624. CSF was created and extensively used to treat digestive diseases and depression for five hundred years. It has been confirmed that CSF exhibits positive effects on the treatment of depression, depression-related diseases and other diseases, including stress-induced hepatic injury, Alzheimer’s disease, and tumors (Kim et al., 2005; Wang et al., 2012; Jia et al., 2017; Zeng et al., 2019; Xiao et al., 2020). Studies of the animal model showed that CSF could inhibit the inflammatory response in different organs, such as the liver, intestine, and brain (Yang et al., 2014; Jia et al., 2018; Liang et al., 2018). In the exploration of the molecular mechanism behind the therapeutic effects of CSF in diseases, researchers found that CSF could regulate various signaling pathways, such as the inhibition of mitogen-activated protein kinase 14 (MAPK14) and glutamate receptor subunit 3 (Gria3) signaling pathways (Liu et al., 2018). Although the digestive tract is the first organ to come after CSF oral administration, the impact of CSF fermentation has been poorly described in general and in the gut micro-environment particularly.

The micro-organisms in the gut represent a biologically active community that lies at the interface of the host with its nutritional environment (Lynch and Pedersen, 2016). The intestine houses trillions of microbial cells, including bacteria, archaea, fungi, and viruses, and the most abundant and prominent component of the normal microbiota is bacteria (Rescigno, 2014; Biedermann and Rogler, 2015). In the adult, the composition of the intestinal microbiota is relatively stable but also dynamically changed in response to different environmental factors. As a consequence, the microbiome plasticity helps the host to rapidly adjust its metabolic and immunologic performances in the face of environmental changes and profoundly influences several aspects of the physiology and metabolism of the host (Candela et al., 2012; Candela et al., 2015). Therefore, the gut microbiome plays an important role in the maintenance of the health status of humans. A healthy gut microbiome is highly diverse, stable and resilient to perturbation, dynamically shaped by external stimulations, and has a high level of redundancy for metabolic pathways (Virgin and Todd, 2011; Lozupone et al., 2012). Interestingly, recent studies demonstrated the concert effects of traditional Chinese medicine and the gut microbiome on the improvement of host health (Zhang et al., 2020).

A wide range of microbial structural components and metabolites directly interact with host intestinal cells to influence nutrient uptake and epithelial health. Both microbial associated molecular patterns (MAMPs) and bacteria-derived metabolites (e.g., short-chain fatty acids (SCFA)) activate various signaling pathways, which dictate the inflammatory tone, energy balance, gut motility and appetite regulation that physiologically connect the gut, liver, muscle, and brain (Nicholson et al., 2012; Ha et al., 2014). SCFA production results from carbohydrate metabolism in the colon and is related to various health effects. The most abundantly produced SCFAs include acetate, propionate and butyrate, which could provide energy and substrates to maintain a healthy body metabolism (Snelson et al., 2021). In contrast, branched SCFA (isobutyrate, isovalerate and isocaproate) result from proteolytic microbial activity, which is associated with the formation of toxic by-products such as p-cresol. Therefore, high branched SCFA production in the colon has been associated with detrimental health effects (Oliphant and Allen-Vercoe, 2019).

The gut barrier consists of immune cells, intestinal bacteria, and epithelial cells held together by intercellular tight junctions. The intact gut barrier acts as a physical and chemical barrier and protects the host from the invasion of toxins, antigens and pathogenic bacteria (Yu, 2018). Gut barrier integrity is important to the maintenance of intestinal homeostasis and thus the host health (Okumura and Takeda, 2018). Numerous diseases were induced by the dysregulation of host-microbiome interactions, such as inflammatory bowel diseases, depression and tumors (Huang et al., 2015; Winter et al., 2018; Zhang et al., 2019). Common to these disorders is the dysregulation of the intestinal epithelial barrier, which is more permeable and results in the initiation of the pathology (Fasano, 2011; Kelly et al., 2015; Stevens et al., 2018; Bertocchi et al., 2021).

When the intestinal barrier function is disrupted, the trafficking of molecules is no longer under control, so that luminal contents may enter the lamina propria and activate the immune system, thereby leading to uncontrolled immune responses (a process known as “leaky gut”), switching a physiological “tolerogenic” inflammation into a detrimental pathological inflammation (Fasano, 2020; Hollander and Kaunitz, 2020). An inflammatory signaling cascade will be initiated with the production of alarm molecules such as pro-inflammatory cytokines (e.g., tumor necrosis factor TNF-α and interleukin (IL-1β) (Li et al., 2010; Wang et al., 2020). These pro-inflammatory cytokines will induce the production of chemokines (e.g., IL-8 and chemokine (C-X-C motif) ligand (CXCL-10)) and adhesion molecules, finally promoting inflammation (Liew, 2003). However, they may also cause tissue disruption and inflammation, leading to the need to resolve the inflammation by the production of anti-inflammatory cytokines, like IL-6 and IL-10. IL-6 possesses both pro- and anti-inflammatory properties (Scheller et al., 2011). IL-6 leads to monocyte/macrophage recruitment *via* activation of monocyte chemoattractant protein (MCP)-1, which promotes the clearance of neutrophils. IL-6 is also able to inhibit the production of pro-inflammatory cytokines such as IL-1. Moreover, IL-6 has a positive effect on the regeneration of the intestinal epithelium and wound healing (Dann et al., 2008). On the other hand, IL-6, together with transforming growth factor (TGF)-β, induces the differentiation of TH17 cells, an important subset of CD4^+^ T cells, which have a key role in host defense against extracellular microbes in mucosal tissues (Morishima et al., 2009).

The Simulator of the Human Intestinal Microbial Ecosystem (SHIME^®^) is an *in vitro* model, which offers an excellent experimental setup to mechanistically study the processes that affect the human gut microbiome. In this study, a down-scaled version of it focusing on the proximal colon allowed to specifically evaluate the capacity of CSF to modulate the gut microbial composition and activity (Van de Wiele et al., 2015). Next, to investigate the impact of CSF fermentation on gut barrier integrity and intestinal immunity, we made use of a previously established co-culture model of intestinal epithelial-like cells (Caco-2 cells) and human monocytes/macrophages (THP1 cells), which mimic the interface between host and gut microbiome (Possemiers et al., 2013). In this study, by combining these two models, we found that CSF exposure stimulated the production of SCFA (acetate, propionate and butyrate) across donors while significantly lowering the production of bSCFA. In terms of gut microbiome composition, CSF thrived the abundance of prebiotics while lowering the abundance of opportunistic pathogenic *Escherichia coli*. Moreover, CSF consisting of Chinese botanical drugs and their microbial metabolites showed protective effects against inflammation-induced intestinal epithelial barrier disruption.

## Materials and Methods

### Preparation of Chaihu-Shugan San Decoction

Chaihu-shugan san formula (CSF) was prepared based on the medical classic Jing-Yue Quan-Shu. It was composed of seven botanical drugs, Radix Bupleuri (Chinese name: Chai Hu, Botanical name: *Bupleurum chinense DC.*, part used: roots, 16 g), Pericarpium Citri Reticulatae (Chinese name: Chen Pi, Botanical name: *Citrus reticulata Blanco.*, part used: pericarps, 16 g), Rhizoma Chuanxiong (Chinese name: Chuan Xiong, Botanical name: *Ligusticum chuanxiong Hort.*, part used: roots, 12 g), Rhizoma Cyperi (Chinese name: Xiang Fu, Botanical name: *Cyperus rotundus L.*, part used: roots, 12 g), Fructus Aurantii (Chinese name: Zhi Qiao, Botanical name: *Citrus aurantium L.*, part used: fruits, 12 g), Radix Paeoniae Alba (Chinese name: Bai Shao, Botanical name: *Paeonia lactiflora Pall.*, part used: roots, 12 g) and Radix Glycyrrhizae (Chinese name: Gan Cao, Botanical name: *Glycyrrhiza uralensis Fisch.*, part used: roots, 4 g).

All crude botanical drugs were obtained from Pharmacy of Traditional Chinese Herbs in Zhejiang Cancer Hospital, Zhejiang, China. The prescription of Chaihu-shugan san was referred to the Pharmacopoeia of the People’s Republic of China (2020 version). To prepare this formula, these raw botanical drugs were immersed in 10-time volume of distilled water for 30min and decocted for 2 h, and the supernatant was collected, filtered with gauze. The concentration of Chaihu-shugan san decoction is 5 g/L.

### CSF Colonic Fermentation

Fecal samples of the healthy donors were collected according to the ethical approval of the University Hospital Ghent (reference number B670201836585) and an informed consent was obtained from all subjects involved in the study. The SHIME^®^ (ProDigest, Ghent, Belgium and Ghent University, Ghent, Belgium) is a dynamic model of the complete human gastrointestinal tract in a controlled *in vitro* setting. The reactor setup consists of five pH-controlled consecutive reactors, which sequentially stimulate stomach, small intestine, ascending colon, transverse colon, and descending colon (Molly et al., 1993). The current study focused on short-term colonic incubations of a single dose of a test compound under conditions representative for the proximal large intestine, using fecal inocula from five healthy donors (3 male, 2 female, aged between 24 and 31) without antibiotic use at least 6 months prior to the inoculation. The fecal samples (five donors, duplicates) were incubated in a sugar-depleted nutritional medium containing basal nutrients of the colon (Possemiers et al., 2013), as described by Marsaux et al. (Marsaux et al., 2020), with or without (blank) the addition of CSF (5 g/L) into the reactor for 48 h incubation at 37°C, under shaking (90 rpm) and anaerobic conditions.

### Microbial Metabolic Activity Analysis

pH value and short-chain fatty acid (SCFA) production, including acetate, propionate, butyrate and branched SCFA (isobutyrate, isovalerate and isocaproate), were measured at the start of the incubation as well as after 48 h incubation (Marsaux et al., 2020). Lactate concentrations were quantified using a commercially available enzymatic assay kit (R-Biopharm, Darmstadt, Germany) according to the manufacturer’s instructions. pH value measurement was performed using a Senseline F410 (ProSense, Oosterhout, Netherlands).

### Microbial Community Analysis

Ten samples were collected at the start (control condition only) and 20 samples after 48 h incubation (all conditions) for microbial community analysis. DNA was isolated from a total of 30 samples according to the method described by Catalayud et al. (Calatayud, 2021). 16S-targeted Illumina sequencing was performed to obtain the microbiota profiling of the colon compartment. DNA quality control, library preparation, and Illumina sequencing of the V3-V4 region were conducted on a MiSeq V3 (2 × 300 bp) at LGC Genomics (Teddington, Middlesex, United Kingdom) using the 341F (5′-CCTACGGGNGGCWGCAG-3′) and 785R (5′- GACTACHVGGGTATCTAAKCC-3′) primers. The detailed procedure of read assembly and cleanup were previously described by Marzorati et al. (Marzorati et al., 2020), which largely derived from the MiSeq SOP described by the Schloss lab (Schloss and Westcott, 2011; Kozich et al., 2013).

### Flow Cytometry of Bacterial Cells

For flow cytometry analysis, 10-fold serial dilutions of luminal samples were prepared in anaerobic Dulbecco’s Phosphate-buffered Saline (DPBS) (Sigma-Aldrich, Bornem, Belgium) and stained with 0.001 mM SYTO24 (Life Technologies Europe, Merelbeke, Belgium) for 15′ at 37°C in the dark. Samples were analyzed on a BD Facsverse (BDBiosciences, Erembodegem, Belgium) using the high-flow-rate setting. Bacteria were separated from medium debris and signal noise by applying a threshold level of 200 on the SYTO channel.

The number of total bacterial cells was used to convert the proportional values obtained with Illumina into absolute quantities by multiplying relative abundances of any population (at any phylogenetic level) in a sample with the total cell counts obtained with fold change of the given sample.

### Cell Culture

The human epithelial Caco-2 cells and human monocytic leukemia THP1 cells were obtained from American Type Culture Collection (HTB-37, LGC Promochem, Molsheim, France) and InvivoGen (Toulouse, France), respectively. Caco-2 cells were maintained in Dulbecco’s Modified Eagle Medium (DMEM) supplemented with 10% (v/v) fetal bovine serum (FBS), and 1% (v/v) penicillin-streptomycin (PS). THP1 cells were cultured in Roswell Park Memorial Institute (RPMI)-1,640 nutrient mixture with 10% (v/v) fetal bovine serum (FBS) and 1% (v/v) penicillin-streptomycin (PS). THP1-Blue™ were obtained from THP1 human monocytes, which were stably transfected with a reporter construct expressing a secreted alkaline phosphatase (SEAP) gene under the control of a promoter inducible by the transcription factor nuclear factor kappa B (NF-κB). Cells were grown in a CO_2_ incubator (37°C, 5% CO_2_).

### Caco-2/THP1-Blue™ Co-culture Model

The co-culture model was established as previously described by Daguet et al., 2016 (Terpend et al., 2013). Briefly, Caco-2 monolayers were cultured for 14 days in 24-well semi-permeable inserts until a functional cell monolayer with a transepithelial electrical resistance (TEER) was obtained. THP1-Blue™ cells were treated with phorbol 12-myristate 13-acetic acid (PMA) that induces the differentiation of the cells into macrophage-like cells in 24-well plates.

The Caco-2-bearing inserts (pore size 0.4 µM) were placed on top of the PMA-differentiated THP1-Blue™ cells (100 ng/ml, 48 h). The apical compartment (containing the Caco-2 cells) was filled with sterile-filtered (0.22 μm) colonic batch suspensions. Caco-2 cells were treated apically with 12 mM sodium butyrate (NaB) (Sigma-Aldrich, St. Louis, MO, United States) as the positive control. The basolateral compartment (containing the THP1-Blue™ cells) was filled with Caco-2 complete medium. Caco-2 cells were also exposed to Caco-2 complete medium in both chambers as control. The duration for the above treatments was 24 h, after which the TEER was measured in all wells (= 24 h time point). After subtracting the TEER of the empty insert, all 24 h values were normalized to their own 0 h value (to account for the differences in the initial TEER of the different inserts) and are presented as a percentage of the initial value.

The basolateral supernatant was then discarded, and cells were stimulated at the basolateral side with Caco-2 complete medium containing ultrapure LPS. Cells were also stimulated at the basolateral side with 500 ng/ml ultrapure LPS (*Escherichia coli* K12, InvivoGen, San Diego, CA, United States) in combination with 1 µM hydrocortisone (HC, Sigma-Aldrich, St. Louis, MO, United States) and medium without LPS (LPS-) as controls. After LPS stimulation, the basolateral supernatants were collected for cytokine measurement [human IL-1β, IL-6, IL-8, IL-10, TNF-α, CXCL10 and MCP-1 by Luminex^®^ multiplex (Affymetrix-eBioscience)] and for NF-κB activity, according to the manufacturers’ instructions. All treatments were done in triplicate. Cells were incubated at 37°C in a humidified atmosphere of air/CO2 (95:5, v/v).

### Statistics

The averages of replicates were calculated and paired two-sided T-tests were performed on obtained averages to verify whether treatment effects were valid across multiple donors.

The experimental controls were presented first in separate plots; these relate to the complete medium control (CM or LPS−), the lipopolysaccharide (LPS+)-treated cells and the Sodium butyrate (NaB) and hydrocortisone (HC) control. Concerning the TEER, the conditions CM and NaB were compared and statistical significance was calculated by using unpaired, two-tailed Student’s t-test. For the immune markers (cytokines/chemokines and NF-κB activity), all conditions (LPS−, LPS + HC and LPS + NaB) were compared to LPS+. Statistical significance was calculated by using one-way ANOVA with Dunnett’s multiple comparisons test against LPS+. The results concerning the colonic batch suspensions presented the average of all donors. All colonic incubations were taken as biological replicates (*n* = 3) in the cell assay.

To evaluate differences in different groups, the treatment of each donor was compared to their non-treated blank control using a two-way ANOVA with Sidak’s multiple comparisons test and are represented by (*). (*), (**), (***) and (****) represent *p* < 0.05, *p* < 0.01, *p* < 0.001 and *p* < 0.0001, respectively. All statistics were performed using GraphPad Prism version 8.3.0 for Windows (GraphPad Software, San Diego, CA, United States).

## Results

### Metabolic Activity

**FIGURE 1:**
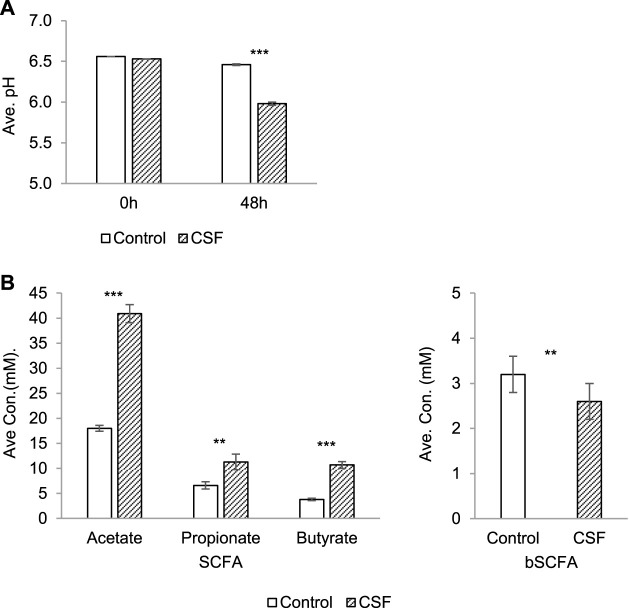
Changes in microbial activity induced by CSF treatment in SHIME. **(A)**. Absolute average pH (±SEM) at the start of the incubation and after 48 h. **(B)**. SCFA and bSCFA production (mM) (±SEM) between 0 and 48 h. Left: the production of SCFA by microbiota, including acetate, propionate and butyrate. Right: the production of bSCFA by microbiota. Two conditions were tested, i.e., a condition with 5 g/L CSF (CSF group) and negative control (control group). Abbreviations: Ave, average (of pH/concentration of all five donors, each donor was measured in triplicates); con, concentration; SEM, standard error of the mean. Significant decreases over control are indicated by asterisk (**: *p* < 0.01, ***: *p* < 0.001, Student’s t-test for pairwise comparisons).

Fermentation activity correlated with ammonium (NH4^+^) and SCFAs profiles (acetate, propionate and butyrate). Monitoring the pH during a colonic incubation provides a good indication of the production of SCFA, lactate and ammonium (NH4^+^). pH decrease was observed in both the negative control and CSF treatment groups upon 48 h incubation (Figure 1A). It showed that the fermentation process proceeded under optimal conditions. The 5 g/L dose was thus optimal to investigate the health-promoting effects of the test product. Interestingly, pH decreases in the CSF treatment (from 6.53 to 5.98) were significantly stronger than pH decreases in the control group (from 6.56 to 6.46). It suggested that the test product CSF was fermented by the colonic microbiota and produced SCFA and/or lactate along the CSF fermentation process (Figure 1A).

The total SCFA level yielded by the test product CSF (67.8 mM) was significantly and dramatically higher than the control (33.3 mM) for each donor (*p* < 0.001), explaining the difference of the pH decreases between the control and CSF treatment groups above. Further investigation showed that the increased SCFA concentrations resulted from stimulatory effects on the 2–3 times higher production of acetate, propionate and butyrate by CSF treatment compared to the negative control (Figure 1B, left). On the contrary, CSF significantly lowered the production of branched SCFA (bSCFA, isobutyrate, isovalerate and isocaproate) in each donor when compared to control. (Figure 1B, right).

### Shifts in Gut Microbiota Community Composition

In this study, we further investigated the effect of CSF on gut microbial communities’ composition of the donors. According to a previous study by Vandeputte et al. (Cheung, 2011), we combine the high-resolution phylogenetic information of the 16S-targeted Illumina with accurate quantification of cell counts *via* flow cytometry to map the community shifts induced by the CSF treatments in large detail. It resulted in the quantitative enumeration of the different taxonomic entities inside the reactors. For community composition of the fecal inocula, the gut microbial communities of the selected donors were mainly represented by three bacterial phyla, i.e., *Actinobacteria* (14.09%), *Bacteroidetes* (37.94%) and *Firmicutes* (47.12%) (Supplementary Figure S1). *Proteobacteria* (0.36%) and *Verrucomicrobia* (0.41%) were present in lower abundances (Supplementary Figure S1). Microbial composition at the family level is provided in Supplementary Table S1, while the most abundant operational taxonomic units (OTUs) are shown in Supplementary Table S2.

**FIGURE 2:**
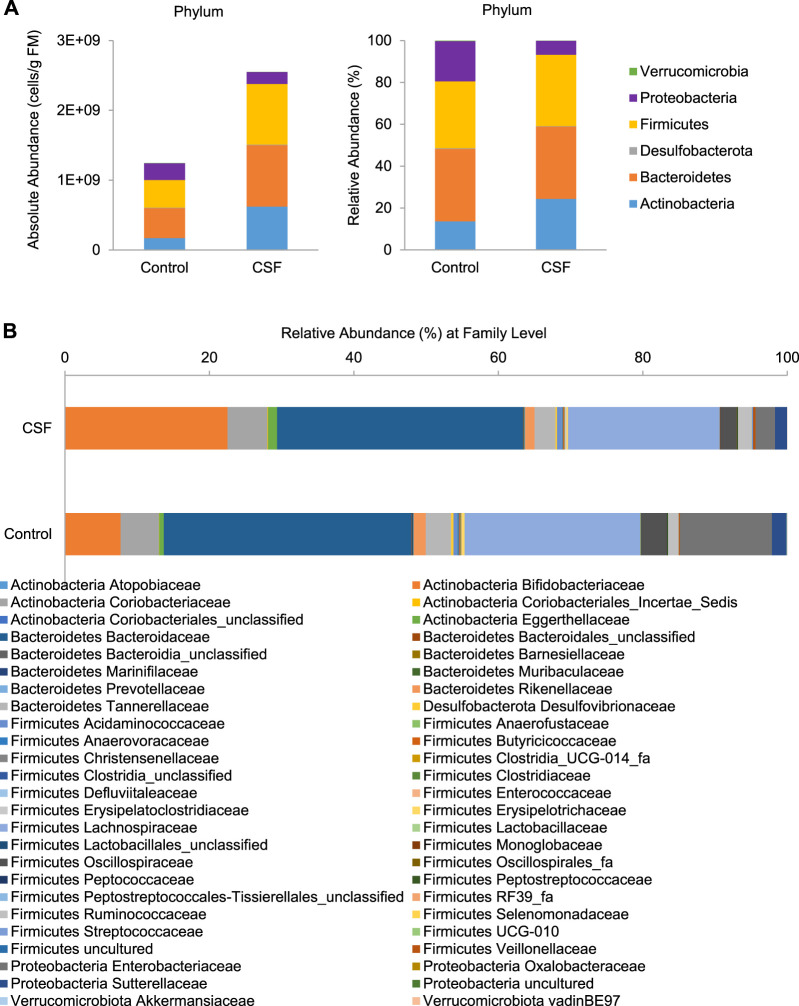
CSF treatment changed the microbial community composition in SHIME. **(A)**. Absolute (expressed as cells/g fecal material (FM), Left) and relative (%, Right) abundances of members of in the fecal samples at the bacterial phylum level. **(B)**. Relative abundances of the bacterial family (%) in the gut microbiome in the fecal samples with or without CSF treatment.

At the phylum level, four main phyla were identified after 48 h incubation in both control and CSF exposure samples: *Actinobacteria*, *Bacteroidetes*, *Firmicutes*, *Proteobacteria*, followed by *Desulfobacterota* and *Verrucomicrobiota* (Figure 2A). Overall, changes were observed after 48 h of CSF treatment in all compartments. Compared with the control group, the CSF treatment promoted an overall increase in gut microbiota more than two times. CSF stimulated the enrichment of *Actinobacteria, Bacteroidetes* and *Firmicutes*, while a significant decrease was visible for the OTUs identified as *Proteobacteria* (Figure 2A, Left). Also, CSF exposure induced a change in gut microbiota community structure. CSF increased the proportion of *Actinobacteria* (from 13.57 to 24.38%) while decreasing the proposition of *Proteobacteria* (from 19.19 to 6.6%) compared to that of the control. CSF didn’t obviously cause a difference in the proposition of *Bacteroidetes and Firmicutes* phyla compared to control (Figure 2A, Right).

At the family level, CSF exposure consistently stimulated a variety of families and OTUs across donors (Figure 2B and Supplementary Table S3). Within *Actinobacteria* phylum, CSF stimulated health-promoting *Bifidobacteriaceae* family, due to significant increases of OTUs identified as *Bifidobacterium* (*Bifidobacterium adolescentis/B. faecale*, from 4.75 to 14.1%), *B. longum* (from 1.02 to 3.38%) and *B.* (*pseudo*)*catenulatum/B. kashiwanohense* (from 0.65 to 2.49%). Further, members of the *Eggerthellaceae* family were also significantly enriched (from 0.80 to 1.55%) by the CSF treatment. Within *Bacteroidetes* phylum, a slight but significant increase was induced by CSF in the relative abundance of *Bacteroidaceae* family due to increases of OTUs identified as *Bacteroides uniformis* (from 2.68 to 3.73%) and *Bacteroides eggerthii* (from 0.42 to 1.47%). Within *Firmicutes* phylum, CSF exposure significantly stimulated the *Acidaminococcaceae, Butyricicoccaceae, Erysipelotrichaceae, Lachnospiraceae Peptostreptococcaceae* and *Streptococcaceae* families, with the most important enrichments including OTUs identified as *Mediterraneibacter faecis/Ruminococcus torques* (from 1.42 to 2.27%), *Blautia faecis* (from 1.61 to 2.08%). Furthermore, within the *Ruminococcaceae* family, OTUs identified as *Faecalibacterium prausnitzii* (from 0.34 to 0.56%) and *Gemmiger formicilis* (from 1.06 to 1.49%) consistently increased. On the contrary, the CSF treatment significantly lowered the abundance of *Enterobacteriaceae* family by decreasing OTU identified as *Escherichia coli/Shigella flexneri/E. fergusonii/S. sonnei/S. boydii/Pseudescherichia vulneris* (from 22.81 to 6.88%), a known pathobiont of the human colon. Therefore, CSF increased the absolute and relative abundance of gut bacteria associated with the production of SCFA (acetate, propionate and butyrate), whereas it led to a decrease in gut bacterial taxa linked to opportunistic pathogens.

### Protective Effect of CSF on Transepithelial Electrical Resistance

To investigate the potential positive effects of CSF and their metabolites produced upon colonic fermentation on gut wall functioning in terms of modulation of a “leaky gut” under inflammatory conditions, we first evaluated the gut barrier integrity in an *in vitro* Caco-2/THP1 co-culture model by the measurement of transepithelial electrical resistance (TEER). Caco-2/THP1 co-cultures were exposed to colonic batch suspensions (with or w/o CSF treatment) for 24 h. The controls [complete Caco-2 medium (CM) and sodium butyrate (NaB, positive control)] behaved as expected (Supplementary Figure S2). The CM control showed an approximately 11% decrease in TEER due to the damage induced by the PMA-activated THP1 cells on Caco-2 monolayers. NaB was able to protect Caco-2 cells from this damage and to maintain and even further significantly increase the TEER of the monolayer (*p*-value < 0.0001, Supplementary Figure S2).

**FIGURE 3:**
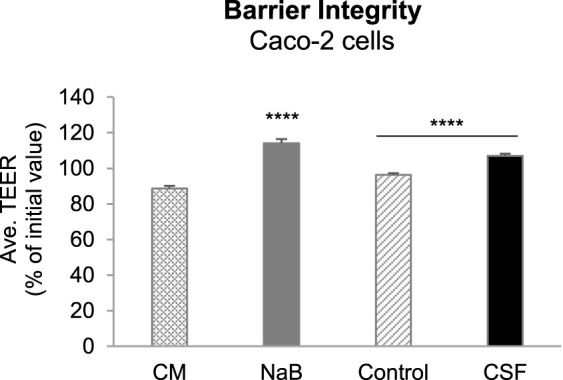
Effect of colonic batch suspensions on transepithelial electrical resistance (TEER) of the Caco-2/THP1-BlueTM co-cultures. Two columns in the left demonstrated the barrier integrity of Caco-2 cells (controls). The Transepithelial electrical resistance (TEER) was measured 24 h after treatment of the Caco-2/THP1-Blue™ co-cultures and each 24 h value was normalized to its corresponding 0 h value and is shown as the percentage of the initial value. The grey dotted line represents 100% (initial value). Two colunms in the right demonstrated that TEER was measured 24 h after pre-treatment of the co-cultures and each 24 h value was normalized to its corresponding 0 h value and is shown as the percentage of the initial value. Data are plotted as mean ± SEM (standard error of the mean). Abbreviations: CM, Caco-2 complete medium; NaB, sodium butyrate; Ave. TEER: average of TEER of all five donors (for each donor, TEER were measured in triplicates). (*) represents statistically significant differences between the control and CSF treatment samples (****: *p* < 0.0001, Student’s t-test for pairwise comparisons).

Exposure of the co-cultures to + CSF colonic suspensions significantly increased the TEER compared to their respective control (-CSF suspensions) (*p*-value < 0.0001, Figure 3). We also observed that colonic suspensions (with or w/o CSF) increased the TEER compared to the CM control. Moreover, the samples (with or w/o CSF) were able to maintain and even further increase the TEER compared to the initial value (Figure 3). Therefore, the fermentation of CSF was sufficient to protect membrane barrier integrity from the inflammation-induced disruption in all five donors tested. It is likely through promoting the abundance of gut microbiota associated with the production of beneficial metabolites, together with the reduction in the abundance of opportunistic pathogenic bacteria, as discussed above.

### The Effect of CSF on the Intestinal Immunity

After 24 h of apical pre-treatment of the Caco-2/THP1 co-cultures with the colonic batch suspensions, the basolateral supernatant was discarded, and the cells were stimulated with LPS. After 6 h of stimulation, the basolateral supernatant was collected to measure cytokines and chemokines secreted in the medium and to determine NF-κB activity.

As expected, LPS was able to increase the secretion of the anti-inflammatory cytokines IL-6 and IL-10 (Supplementary Figure S3A) and of the pro-inflammatory cytokines IL-1β and TNF-α (Supplementary Figure S3B) and chemokines CXCL10, IL-8 and MCP-1 (Supplementary Figure S3C). Hydrocortisone (HC), being a corticosteroid, acted as a broad immunosuppressant by dampening LPS induced cytokines and chemokines (Supplementary Figures S3A–C). In contrast, sodium butyrate (NaB) showed marker-dependent effects. NaB showed clear selective post-transcriptional inhibitory activities on some immune mediators. More specific, NaB selectively increased LPS-induced IL-6 and IL-10 secretion (involved in immune homeostasis) (Supplementary Figure S3A), while NaB selectively inhibited LPS-induced IL-1β and TNF-α (pro-inflammatory cytokines) (Supplementary Figure S3B) and CXCL10, IL-8 and MCP-1 (chemokines involved in the recruitment of immune cells) (Supplementary Figure S3C) secretion.

**FIGURE 4:**
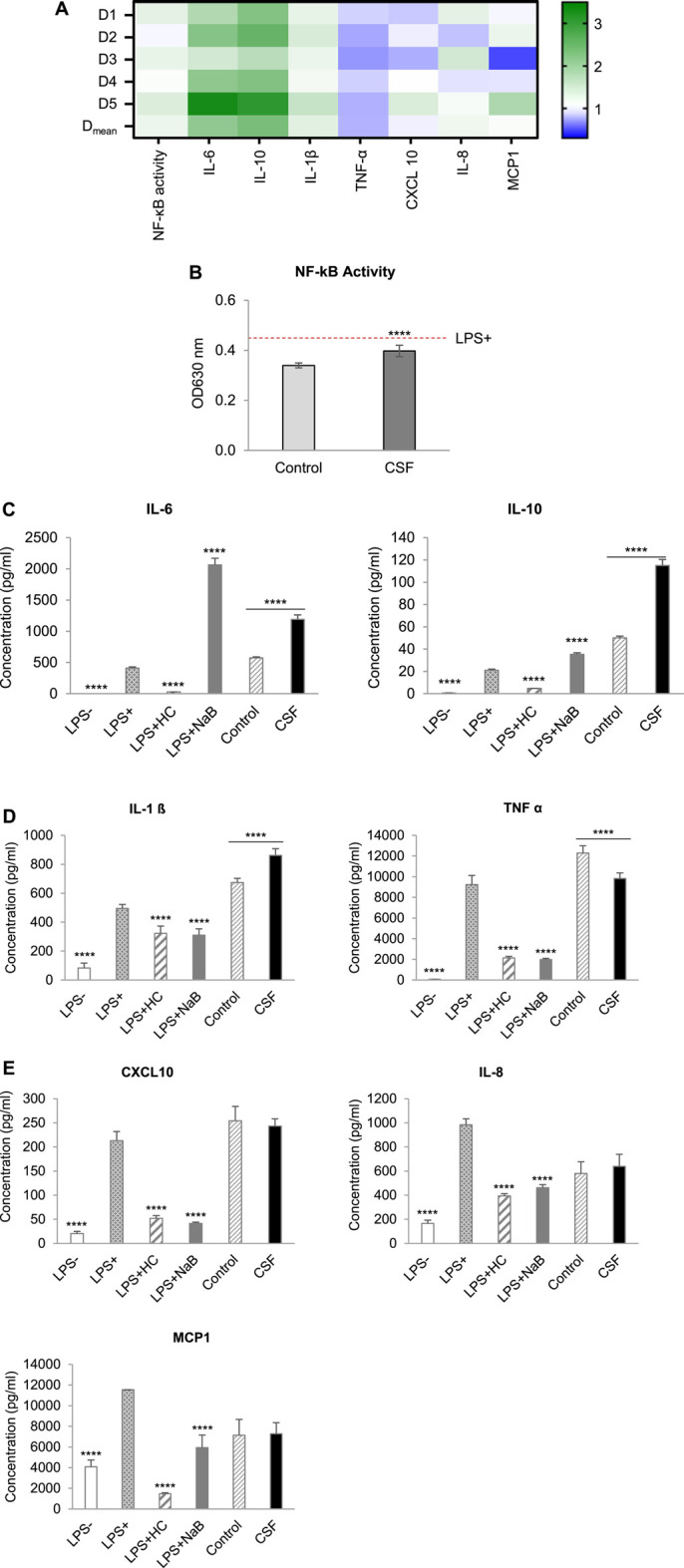
Effect of CSF on NF-kB Activity, cytokine and chemokine release of PMA-treated THP1-Blue™ cells after LPS stimulation in the Caco-2/THP1-Blue™ co-culture model. **(A)**. Heatmap of an overview of the fold changes of NF-kB Activity, cytokine and chemokine release induced by the CSF treatment comparatively to the blank control. Cell experiment results for the treatment colonic batch suspensions normalized to the blank control colonic batch suspensions. Values close to 1 (no change from control) are depicted in white or light blue; values above 1 (treatment higher than control) are depicted in green; values below 1 (treatment lower than control) are depicted in blue. Color intensity is proportional to the degree of change. There are five donors in total: D_1_, D_2_, D_3_, D_4_ and D_5_. Each donor was measured in triplicates. D_mean_: the mean of the absolute value. **(B)**. NF-κB activity of THP1-Blue™ cells, **(C)**. The secretion of the anti-inflammatory cytokines IL-6 and IL-10. **(D)**. The secretion of the pro-inflammatory cytokines IL-1ß and TNF-α, **(E)**. The secretion of the pro-inflammatory chemokines CXCL10, IL-8 and MCP-1, were measured 6 h after LPS treatment on the basolateral side of the Caco-2/THP1-Blue™ co-cultures after pre-treatment of the apical side for 24 h by the colonic batch suspensions with or without CSF. **(C–E)**: Four columns in the left, basolateral secretion, two columns in the right, the secretion upon CSF treatment. Data are plotted as mean ± SEM. (*) represents statistically significant differences between the blank and treatment samples (****: *p*-value < 0.0001, Student’s t-test for pairwise comparisons). Abbreviations: LPS, lipopolysaccharide; LPS−: cells treated with complete medium (no LPS); LPS+: LPS treated cells; HC, hydrocortisone; NaB, Sodium butyrate; SEM, standard error of the mean. There are five donors in total. Each donor was measured in triplicates.

The effects of colonic batch suspensions on NF-κB activity, cytokine and chemokine release in the Caco-2/THP1 co-culture system were reported in Figure 4 (The normalized values of markers in each individual donor and their average value with statistical analysis were displayed in Figure 4A and Figures 4B–E, respectively). On average, colonic batch suspensions with or without CSF all decreased the NF-κB activity compared to the LPS + control (Figure 4B). Furthermore, on average, fermentation of CSF showed a significant increase in NF-κB activity compared to the control (*p*-value < 0.0001).

By exploring the inflammatory effects induced by CSF fermentation, the secretion of anti-inflammatory cytokines IL-6 and IL-10, as well as the pro-inflammatory cytokine IL-1β and TNF-α, were tested. With or without CSF added, colonic batch suspensions all increased the secretion of the anti-inflammatory cytokines IL-6 and IL-10, compared to the LPS + control (Figure 4C). In addition, CSF treatment significantly increased IL-6 and IL-10 levels compared to their respective blank controls. It confirmed that fermentation of the test product CSF showed strong beneficial anti-inflammatory effects by significantly increasing IL-6 and IL-10 secretion in all donors (Figures 4A,C, *p*-value < 0.0001). Next, the pro-inflammatory cytokine IL-1β and TNF-α were examined. Colonic batch supernatants with or without CSF all increased the LPS-induced secretion of the pro-inflammatory cytokine IL-1β, compared to the LPS + control (Figure 4D, Left). Furthermore, incubation with CSF significantly increased IL-1β secretion, compared to their respective blank control in all donors (*p*-value < 0.0001). Concerning pro-inflammatory cytokine TNF-α, there’s a significant decrease in secretion upon treatment with CSF compared to that in control (Figures 4A,D Right, *p*-value < 0.0001). To conclude, in all donors, colonic fermentation of CSF resulted in increased pro-inflammatory cytokine IL-1β levels, while in contrast, secretion of pro-inflammatory cytokine TNF-α was reduced.

The CSF effect on the recruitment of leukocytes was investigated through the examination of chemokines CXCL10, IL-8 and MCP-1. When looking at the LPS-induced secretion of chemokines CXCL10 and MCP-1, colonic supernatants increased CXCL10 secretion while decreasing MCP-1 secretion (Figure 4E). Interestingly, CSF treatment revealed donor-dependent effects referring to the secretion of chemokines CXCL10 and MCP-1 (Figure 4A). In donor D_3_, a significant decrease in CXCL10 and MCP-1 levels was shown (decreased 22.5 and 50.4%, respectively), compared to their respective controls. In contrast, a significant increase was shown in donor D_5_ (increased 36.2 and 78.4% in CXCL10 and MCP-1 levels, respectively). CSF treatment did not affect CXCL10 and MCP-1 levels in donors D_1_, D_2_ and D_4_ (increased or decreased <20%). When looking at the average of all donors, after CSF treatment, CXCL10 and MCP-1 levels were not significantly affected, compared to the control (Figure 4E, *p*-value < 0.05).

Concerning chemokine IL-8 levels, colonic supernatants decreased LPS-induced secretion in both CSF treatment and the CSF negative control groups, compared to the LPS + control [(Figure 4E). A significant increase in IL-8 levels was seen in donor D_1_ and D_3_ compared to its control (increased 25.9 and 43.5%, respectively). However, CSF treatment did not affect IL-8 levels in all other donors (increased or decreased <20%]. This was reflected on the average of all donors, for which treatment and control showed comparable IL-8 levels. In conclusion, colonic fermentation of CSF, consisting of Chinese botanical drugs, showed donor-dependent effects on CXCL10, IL-8 and MCP-1 secretion.

In summary, CSF was well fermented by the gut microbiota, which was observed from the marked pH decrease during the incubation as the product stimulated the production of SCFA (acetate, propionate and butyrate) while significantly lowering the production of bSCFA. In terms of community composition, CSF fermentation stimulated a broad spectrum of health-related species, such as *Bifidobacterium*, *Bacteroidaceae*, *Rikenellaceae* and *Acidaminococcaceae*, which are potent SCFA producers. At the same time, CSF exposure lowered the abundance of opportunistic pathogenic *Escherichia coli*. Interestingly, colonic fermentation of CSF showed protective effects on inflammation-induced intestinal epithelial barrier disruption in. Also, CSF treatment showed beneficial, interesting immuno-modulatory effects. CSF showed anti-inflammatory properties by highly increasing anti-inflammatory cytokines IL-6 and IL-10 and reducing pro-inflammatory cytokine TNF-α. Finally, the overall effects on chemokines CXCL10, IL-8 and MCP-1 were rather mild and more donor-dependent. Therefore, the study using a dynamic *in vitro* simulator model of the human colon showed that CSF improves gut health through the promotion of health-related gut bacteria, protective effect on intestinal barrier, as well as anti-inflammatory properties in health donors. Although a short-term simulation of the human colon used here can exactly mimic the different colon regions of human beings, the observations got from this *in vitro* model may still have some deviations from the real endogenous changes induced by CSF on gut microbiomes, barrier and immunity. Thus *in vitro* experiments are necessary for the validation of the observed changes.

## Discussion

In this study, the effect of traditional Chinese medicine–Chaihu Shugan Formula (CSF), consisting of several Chinese botanical drugs like Bupleurum, on the gut microbiome, gut barrier and intestinal immunity was evaluated using *in vitro* models–a short-term simulation of the human proximal colon combined with Caco-2/THP1 cell co-culture model. We demonstrated that CSF exposure modulated the gut microbiota composition, which significantly increased the abundance of health-promoting bacteria and subsequently resulted in increased production of the beneficial SCFA metabolites. Also, CSF and its metabolites showed a protective effect on gut barrier integrity. Moreover, this CSF-microbiome-related interaction decreased the secretion of certain pro-inflammatory biomarkers while increasing the secretion of some anti-inflammatory biomarkers. To the best of our knowledge, this is the first study evaluating the beneficial colonic microenvironment created by CSF and its metabolites in healthy people using *in vitro* models.

A significant decrease in pH was observed throughout the CSF exposure in relation to the control. The pH value is used to evaluate the production of SCFA, lactate and ammonium (NH_4_+) during a colonic incubation. A pH decrease during colonic incubation could be due to various factors, but mainly caused by three factors, acting singly or in combination: 1) stimulatory effects on the production of acidic metabolites (SCFA and/or lactate) upon substrate fermentation, which is the most probable cause of pH decrease during colonic incubation. 2) a fall of ammonia (Vince et al., 1973), which usually resulted from proteolytic fermentation or urea hydrolysis by bacteria. The decline of colonic ammonia is considered beneficial to a person’s health. The gut ammonia could be absorbed into the circulating system and the high ammonia level in the blood is toxic. Ammonia is an important factor in the pathogenesis of hepatic encephalopathy (Romero-Gómez et al., 2009). It is also involved in the colonic carcinogenic response by creating a tumor-favorable environment in the colon (Fung et al., 2013). 2) a decrease in the conversion of stronger acids into weaker acids through cross-feeding (for instance, acetate/lactate-to-propionate/butyrate conversion).

CSF fermentation significantly increased short-chain fatty acids (SCFA) secretion and concentrations *in vitro* model by the increased production of acetate, propionate and butyrate. SCFA is essential for the maintenance of gut and metabolic health by various effects (Koh et al., 2016; Blaak et al., 2020). Acetate can be used as an energy source for the host and as a potential substrate for lipid synthesis in the body; propionate reduces cholesterol and fatty acid synthesis in the liver (beneficial effect on metabolic homeostasis). Butyrate on the other hand, is a major energy source for colonocytes and induces differentiation in these cells (related to cancer prevention). At the same time, CSF significantly inhibited the production of bSCFA. Like ammonium, branched SCFA production results from proteolytic microbial activity, which is a process that promotes the formation of toxic compounds that have the potential to damage the host epithelium and cause inflammation (Rios-Covian et al., 2020). Therefore, an increase of acetate, propionate and/or butyrate accompanied with a decrease of bSCFA demonstrated the positive effects of CSF fermentation on the host metabolic health.

Acetate can be produced by many different gut microbes (including amongst others *Bifidobacterium*, *Bacteroides* and *Lactobacillus spp.*) and is a primary metabolite produced from the fermentation of prebiotic substrates. Propionate can also be produced by different gut microbes, with the most abundant propionate producers being *Bacteroides spp.* (phylum = *Bacteroidetes*), *Akkermansia muciniphila* (phylum = *Verrucomicrobia*) and *Veillonellaceae* (phylum = *Firmicutes*). Butyrate is produced by members of the *Clostridium* clusters IV and XIVa (phylum = *Firmicutes*). In a process called cross-feeding, these microbes convert acetate and/or lactate (along with other substrates) to the health-related butyrate. In our study, the CSF and its metabolites stimulated the enrichment of *Actinobacteria*, *Bacteroidetes* and *Firmicutes*, while with a significant decrease in *Proteobacteria*, which is consistent with the alteration in the production of SCFA and bSCFA. It indicated that CSF fermentation thrived the growth of health-promoting gut microbes while inhibiting the harmful bacteria, which subsequently promoted the production of SCFA but reduced the secretion of bSCFA.

Interestingly, CSF and its microbial metabolites significantly enhanced gut barrier integrity. The intestinal epithelial barrier is formed by intercellular tight junctions, a complex protein-protein network that mechanically links adjacent cells and seals the intercellular space. Previous studies demonstrated that the intestinal epithelial barrier controlled the equilibrium between immune tolerance and immune activation and so it has a prominent role in “leaky gut” pathogenesis (Antonini et al., 2019). Within the gut, chemical, mechanical or pathogen-triggered barrier disruption led to the influx of bacteria, microbial metabolites and by-products from the lumen into the lamina propria. This will activate the immune system, which will switch from a physiological “tolerogenic” inflammation into a detrimental pathological inflammation. The disruption of gut barrier integrity also could lead to microbiota dysbiosis, and subsequently promote the development of various diseases, including inflammatory bowel disease and colorectal cancers (Yu, 2018). Thus, the CSF-induced enhancement of gut barrier integrity can be considered as a health benefit for the maintenance of intestinal immune homeostasis and the host health, which was confirmed by the health-promoting alteration in gut microbiome composition as well as the anti-inflammatory effect induced by CSF exposure *in vitro* model.

Notably, in this study, through our particular setup, the colonic suspensions are brought in contact with the apical side of the co-cultures (Caco-2 cells). The effects observed on the basolateral chamber (where the THP1 cells reside) are then mediated indirectly by signals produced by the Caco- 2 cells and/or by the transport of micro- and macro-molecules. The unique aspect of this approach resides in the fact that it allows evaluating the effect induced by CSF and the fermentation-derived metabolites produced by the gut microbiota during the digestive steps but not only by the pure CSF product. The combined *in vitro* models could directly and simply have evaluated the CSF effects on the colon microenvironment but not been affected by other organs and biological activities. However, a limitation has also been brought because of the *in vitro* experiments used here. *In vitro* models may not reflect the real endogenous effects induced by CSF on gut microbiomes and gut immunity and thus a clinical trial is essential to confirm the observed results.

## Data Availability

The data presented in the study are deposited in the Sequence Read Archive (SRA) repository, accession number PRJNA807003.
